# Switchgear Digitalization—Research Path, Status, and Future Work

**DOI:** 10.3390/s22207922

**Published:** 2022-10-18

**Authors:** Nediljko Kaštelan, Igor Vujović, Maja Krčum, Nur Assani

**Affiliations:** Faculty of Maritime Studies, University of Split, Ul. Ruđera Boškovića 37, 21000 Split, Croatia

**Keywords:** switchgear digitalization, preventive maintenance, condition monitoring, data-driven maintenance

## Abstract

To keep pace with global energy efficiency trends and, in particular, emission reduction targets in the maritime sector, both onshore and maritime power distribution systems need to be adapted to the relevant new technologies and concepts. As an important link in the distribution chain, medium-voltage switchgear (MV) is expected to be stable and reliable while operating as efficiently as possible. Failures of MV equipment, while rare because the equipment must be safe to handle and use, have far-reaching consequences. The consequences of such failures, whether to the shore or marine power system, present risks to the entire power plant, so an accurate assessment of equipment condition is required to identify potential failures early. The solution is an emerging concept of digital switchgear, where the implementation of sensor technology and communication protocols enables effective condition monitoring, and the creation of a database that, when combined with machine learning algorithms, enables predictive maintenance and/or fault detection. This paper presents, step by step, the previous challenges, the current research (divided into predictive maintenance, condition monitoring, and fault detection categories), and the future directions in this field. The use of artificial intelligence is discussed to eliminate the disadvantage of manually interpreting operational data, and recommendations for future work are formulated, such as the need to standardize test procedures and data sets to train and compare different algorithms before they are used in practice.

## 1. Introduction

According to the reports of the International Energy Agency (IEA), despite the destabilization of global energy consumption due to the pandemic, electricity demand will reach pre-pandemic levels in 2022, and continue to increase [1]. Analogous to the increase in demand on land, the demand for electricity on board ships will also increase, due to the ongoing electrification of ships and a growing number of electricity-based technologies [2,3,4], the implementation of which contributes to the International Maritime Organization’s (IMO) goal of reducing greenhouse gas emissions by 2050 [5] while increasing energy efficiency. Consequently, the increasing demand requires improvements in two areas: power generation and power distribution. It should be noted that, due to functional similarities, the advances in land-based distribution systems can also be considered as improvements in shipboard distribution systems, focusing on increasing efficiency and reliability, as it is an independent, closed system that can be considered as an islanded microgrid at sea [6]. The inefficiency of the power distribution system, if not at an appropriate level, can outweigh the efficiency of the power generation system, which is why it is important to link the improvements in both areas. Switchgear, as an essential component of the distribution system, is used to control, protect, and isolate power systems, as well as disconnect equipment for repair, maintenance, and testing purposes. Considering the role of switchgear and the usually high current flow loads to which the equipment is subjected, especially in medium voltage networks, maintenance strategies are required, the optimization of which would be of great benefit for real-time condition monitoring and diagnosis [7]. The procedures now mostly rely on predefined maintenance intervals and observing the thermal effects by means of manual IR temperature measurements. With this in mind, several studies and experiments have been conducted to leverage the recent beneficial results of deep learning-based methods and Internet-of-Things (IoT) to create solutions for efficient power distribution monitoring. In [8], deep learning was used to diagnose power quality disturbances in electric power systems to reduce existing delayed actions based on mathematical calculations. The implementation resulted in over 99% accuracy using simulated data. Another study, [9], proposed a high-throughput, low-latency deep-learning-based approach in an edge device to detect high-impedance faults on overhead power lines in real time. The approach was validated under laboratory conditions and showed reduced detection latency and high detection accuracy of 96.67%. Additionally, in [10], an intelligent device was presented for monitoring polluted high voltage insulators in overhead transmission lines to prevent power outages before they occurred. The device was tested under laboratory conditions and achieved an accuracy of 91.66%. Although the advantages of such a system are numerous (simplicity, low cost, ease of use, etc.), the limitation lies in the problems of the internet network, and the adjustment of the reference current leakage depending on the pollution of the area where the lines are installed. In order to quantify the research focus in this area, the highly regarded Web of Science (WoS) database was searched for studies on switchgear digitization and intelligent fault diagnosis techniques from the last decade. With respect to the topic of this paper, the core collection of WoS, or more specifically, the Science Citation Index Expanded (SCIE) and the Social Science Citation Index (SSCI), were selected. In addition, only articles and reviews were considered, and publication years were limited from 2011 to 2021. The selection of keywords was of great importance, and due to the wide variety of connotations for the searched topic, it was difficult to include all relevant keywords. In this paper, the search format “object” (e.g., switchgear, grid, etc.) + “interest” (e.g., diagnostics, digitization, etc.) was used, and the query is as follows:

(1) $$\mathrm{TI}\;=\;\{\begin{array}{c} \left(\mathrm{grid}\;\mathrm{OR}\;\mathrm{switchgear}\;\mathrm{OR}\;\mathrm{power}\;\mathrm{system}\;\mathrm{OR}\;\mathrm{electrical}\;\mathrm{equipment}\right)\\ \mathrm{AND}\;(\mathrm{smart}\;\mathrm{OR}\;\mathrm{digital}\;\mathrm{OR}\;\mathrm{fault}\;\mathrm{OR}\;{\mathrm{diagn}}^{*}\;\mathrm{OR}\;\mathrm{machine}\;\mathrm{OR}\;\mathrm{learning}\;\mathrm{OR}\;\mathrm{twin}\;\mathrm{OR}\;\mathrm{IoT}\\ \mathrm{OR}\;\mathrm{predictive}\;\mathrm{OR}\;\mathrm{maintenance}) \end{array}\}$$

**Figure 1:**
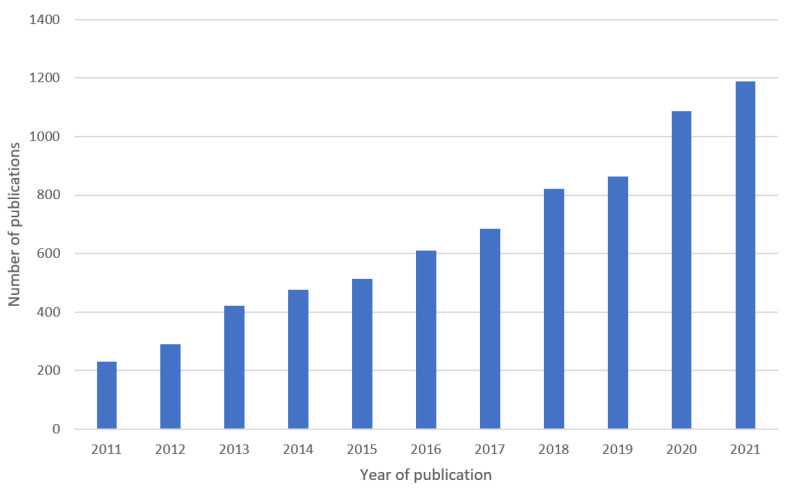
Number of publications related to the research topic in the past decade.

**Figure 2:**
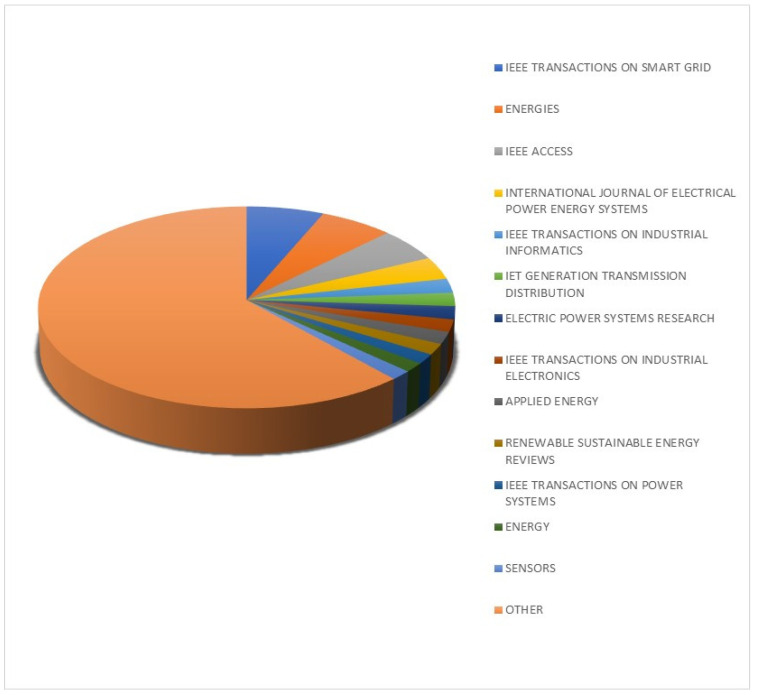
Major publishers in the research topic.

After excluding unrelated topics such as economics, psychology, etc., a total of 7187 documents were collected. The chart in Figure 1 shows the total increase in published papers by year, while Figure 2 shows the major publishers.

Overall, the concept of online condition monitoring using digital sensors and communication protocols, or the concept of digital switchgear (a switchgear in which the information on equipment status, current and voltage measurements, and commands are reliably transmitted over a common communication bus, while the condition monitoring and diagnostic information of the switchgear and its equipment is also digitally available for advanced analysis [11]), is the most practical tool for maximizing the efficiency of maintenance strategies and all the resulting benefits of digitalization [12]. However, it also comes with disadvantages [13], such as the need for solid knowledge of communication networks and adaptation of personnel to digital measurements (software analysis). Another problem posed by the digitalization of the power grid, and in particular by its integration with the IoT, is vulnerability to cyber-attacks due to the increasing reliance on communication networks to support physical process control, a problem that has been recognized and for which a large number of studies already exist, some of which are [14,15,16]. Given the potential importance of distribution system digitization, this scoping review summarizes and appropriately categorizes substation digitization research based on the steps that preceded the emergence of the concept of digital substations. It concludes with current relevant applications, benefits, and recommendations for future work to address the most common problem of forming a framework for direct the comparison of developed algorithms for use in maintenance purposes, and hopefully create a baseline database for switchgear digitalization to guide future research. The guiding principle of this scoping review paper is based on the consideration that: “The knowledge of how a system started can provide a better understanding of what it is and why it is significant” [17]. The structure of the paper is as follows:

- Section 2 describes previous optimization efforts in switchgear and explains the need to update metering equipment in switchgear.
- Section 2.1 describes the implementation of sensors and the resulting benefits.
- Section 3 describes the communication protocol that allows the switchgear to meet the above definition of a digital switchgear.
- Section 4 describes the different measurements and sensor types.
- Section 5 describes the use of the data manipulations collected by the sensors in terms of fault detection, condition monitoring, and predictive maintenance.
- Section 6 gives a discussion.
- Section 7 gives the conclusions from this research.

## 2. The Initial Situation

The longest standing method for the development of switchgear was based on optimizing the physical and electrical properties of the components that make up the current path. Some examples of previous and still ongoing optimization efforts of the busbar configuration are achieved either by geometric distribution to reduce losses [18], reduction of electrodynamic forces in case of faults [19], and analysis of various other factors that contribute to increased energy efficiency, reduction of dimensions, weight reduction, etc. The measurement equipment was limited to conventional instrument transformers (IT) and “hardwire” transmission of measurement data to analog panel meters and protection relay circuits. Considering the disadvantages of these devices, such as high losses, large weight, lower accuracy, and various hazards resulting from their operating principles [20], the equipment had to be revised and updated or replaced by more effective solutions. The progress in this field, with respect to MV switchgear, is mainly due to the development of microcontroller-based protection relays, i.e., intelligent electronic devices (IEDs) [21].

### 2.1. Replacement of Outdated Measurement Components

As mentioned, the development of microcontroller-based IEDs, which do not require the high-level outputs of ITs, enabled the implementation of sensors in switchgear. The operating principles of these sensors, which replace traditional ITs in measuring voltage or current, are not new, but the area of implementation is. Some of the most popular solutions offered to replace obsolete components are:

- resistive or capacitive voltage dividers as voltage sensors;
- Rogowski coil as current sensor;
- current sensor with non-saturable magnetic core.

The general advantages offered by these sensors compared to conventional ITs are:

- non-saturable, since no iron core is used;
- high degree of accuracy;
- increased personnel safety (low secondary voltages);
- small size and weight;
- wide dynamic range;
- environmental friendliness, as less raw material is used;
- no damage caused in case of overload.

A more detailed comparison of conventional ITs with current sensors and conventional ITs with voltage sensors is presented in [22]. 

To illustrate the energy savings from the use of sensors, a typical panel model with conventional current transformers is compared to a model with implemented current sensors in [23]. The selected switchboard consists of fourteen panels:

- two incoming feeders;
- twelve outgoing feeders.

The results show that energy consumption can be significantly reduced by the use of sensors, especially in the long-term comparison, contributing to the worldwide efforts to reduce energy consumption. In view of the above comparison, it is obvious that replacing IT with sensors represents a major advance in the field.

## 3. The Broadening of the Perspective

As mentioned earlier, the predominant transmission of measurement data in the IT era was to hardwire the output to the desired point of data manipulation. This severely limited the scope of data transmission and the ability to use the data for a larger purpose. Sensors, with their low-power signals, simplified the transmission of measured data, but the broader application of measured values at the switchboard level, or between multiple IEDs and a central management station, required the development of a standardized communications protocol. The development of the IEC-61850 communication protocol can be considered the foundation for the switchgear digitization process. As explained in [24], “IEC 61850 is an Ethernet-based standard for substation communication. It was established in 2003 by International Electrotechnical Commission’s (IEC) Technical Committee 57 (TC57). This standard takes advantage of a comprehensive object-oriented data model and the Ethernet technology by bringing in great reduction of the configuration and maintenance cost. The architecture of IEC 61850 makes it suitable for domains besides substation automation (SA) and smart grid. The standard achieves both vertical and horizontal communications, which are needed for optimum performance inside the SCADA (Supervisory Control and Data Acquisition)”. The basics of the protocol configuration are described in [25], and its implementation is explained in [26] to automate substation operations and address the impact of communication delays on the automated system during peak loads. In addition, according to [27], the implementation of the protocol (together with the included GOOSE and SCADA systems) in a power distribution system will increase the system’s reliability by 1.5–2 times, and power losses by 10% or more. Finally, with the endorsement of the above protocol, the way has been opened for achieving what is defined as digital switchgear.

## 4. Expansion of the Types of Measurement Sensors

Considering the various phenomena that manifest the symptoms of failure modes in switchgear, such as the increase in temperature as the electrical connections deteriorate, the effect of increased humidity on the deterioration of insulation, and the development of partial discharges, which are also indicative of various deteriorations of switchgear operation, the need to measure various parameters is obvious. For example, a study was performed to analyze failure phenomena and characteristics, while also highlighting the complex relationships among cause, effect, and failure modes in switchgear [28]. 

Based on the parameters they measure, sensors can be divided into the following categories:

- temperature sensors;
- humidity sensors;
- partial discharge sensors.

### 4.1. Temperature Measurements

A temperature rise at the electrical connections, as shown by several examples in [29,30], can be an indication within switchgears of deterioration of the contact surfaces and/or loosening of the tightening mechanisms (nuts and washers) of connections to copper busbars. Currently, various design solutions are being developed to alleviate the above problem; the most common being [31]:

- surface acoustic wave (SAW) temperature sensors;
- infrared (IR) temperature sensors;
- IR window implementation;
- fiber optic sensors.

SAWs measurement technology offers the ability to passively measure temperature, with the biggest advantage being that it is wireless. The sensors themselves contain an inter-digitized transducer (IDT) that is interrogated wirelessly. The major advantage over active sensors, especially if they are not easily accessible, is that they do not require battery replacement. The sensitive element is a piezoelectric quartz substrate housed on a hermetically sealed stainless-steel base. The sensor can be securely attached to the surface to be measured with a screw [32].

The most popular IR solutions used in switchgear assemblies are the separate IR temperature sensors housed in the switchgear [33] or manual measurements using an IR camera through a IR window [34], built into the switchgear enclosure and pointed at the spot to be measured.

### 4.2. Humidity Measurements

The SAW temperature sensors mentioned above can also be used as humidity sensors [35]. The importance of humidity measurements lies in the problem that humidity accelerates the degradation of materials and increases the risk of partial discharges [36,37].

### 4.3. Partial Discharge (Arc) Measurements

Similar to the importance of a temperature rise in switchgear, the occurrence of a partial discharge is a sign that stable operation of either the switchgear or a major component has been compromised. Depending on the magnitude of the partial discharge, the consequences can lead to critical failures if not detected as soon as possible, underscoring the importance of measurement technology. Due to the nature of the partial discharge phenomenon, several paths can be taken to predict or detect it, and the detailed explanation and comparison of different possibilities can be found in [38]. 

With the different types of data that can be collected by different sensors, the question of the influence of sensor accuracy or measurement uncertainty arises when they are translated into different methods of measurement data manipulation (described in the following chapter). Since every measurement is subject to inaccuracies, repeated measurements lead to fluctuations, which are mostly caused by random effects (temperature, humidity, air pressure variations, etc.). Consequently, the measurement uncertainty caused by sensors significantly affects the accuracy of machine/deep learning models, especially in safety-critical applications. To combat this recognized problem, several studies have been conducted with positive results, such as [39], which is limited to supervised machine learning regression techniques, and [40], which proposed a method for calibrating uncertainty prediction for regression tasks. Ultimately, the data obtained from the sensors, considering the measurement uncertainty, combined with IEDs using the IEC 61850 communication protocol, provided the basis for defining digital switchgear.

## 5. Applications of Measurement Data Manipulation

**Figure 3:**
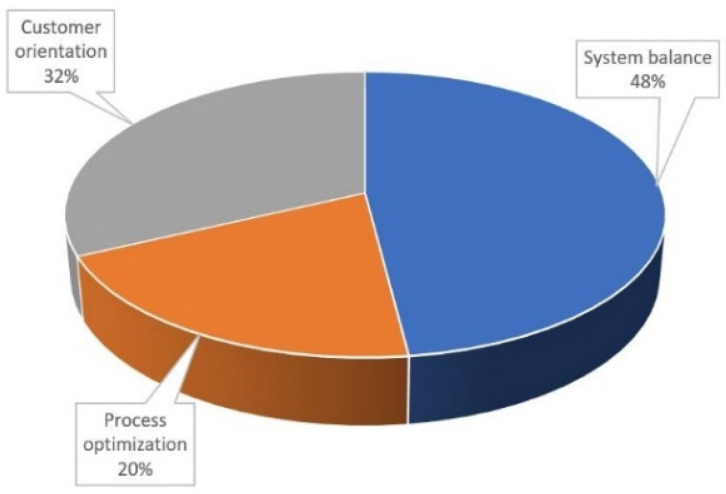
Analysis of digital applications in smart networks.

The digital applications used in the “smart grids”, an intertwined term used to describe the digital distribution network, i.e., all distribution components including the switchgear, as investigated in [41] in 2019, highlight and divide the research conducted in the field of digital applications in condition monitoring and predictive maintenance in distribution networks and its included subcomponents. The results are presented in Figure 3. 

**Figure 4:**
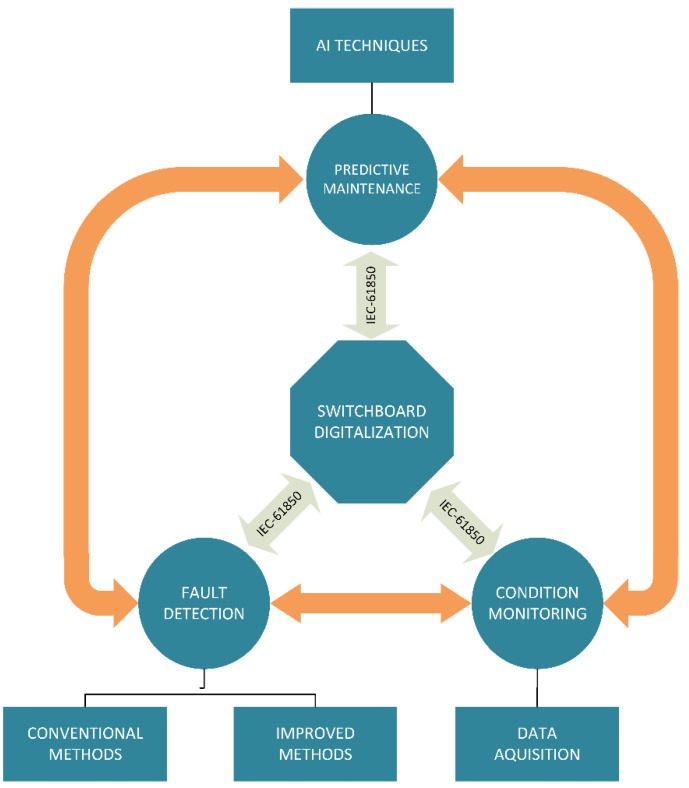
Interconnection of switchboard digitalization activities.

Where, in system balance real time condition monitoring and anomaly detection/failure localization, related papers make up 32%, respectively, process optimization digital twin and predictive maintenance-related papers make up 75%. The significant increase of research in the field in the last two decades has seen an exponential growth of predictive maintenance-related papers, while machine learning-infused predictive maintenance papers present around 44% of all related publications between 2020 and 2021 [42]. The processes, such as predictive maintenance, fault detection, and condition monitoring, that utilize the measured data (directly or indirectly) are interconnected as each process can either be improved or makes use of the bidirectional flow of information, as shown in Figure 4.

In the above figure, the orange arrows depict the bidirectional flow of information, and condition monitoring is utilized in the following scheme for data acquisition that is further processed for use with the purpose of fault detection or predictive maintenance. Conversely, the results of the analysis of predictive maintenance or the detection of faults can improve the equipment monitoring by providing data as to optimize the location of sensors or add/remove different types of sensors. In the following subsections, the various applications in each category will be exemplified, but due to significant interconnection they are not always easy to differentiate. 

### 5.1. Condition Monitoring

The process of monitoring multiple conditions of an object (temperature, vibrations, partial discharges, etc.) that are considered to be health indicators, in order to identify changes in operation that indicate the development of a possible fault, is called condition monitoring (CM). It is a significant part of predictive maintenance, as the acquired data is used to optimize and perform scheduled and preventive actions in order to prevent asset failure and unwanted downtime. In the case of a marine switchgear, condition monitoring (and the implied predictive maintenance and fault detection processes) is a viable tool for increasing operational safety, efficiency of the equipment, and reducing major failures risk and the accompanying possibility of arcing faults during maintenance and repair operations that are described in [43]. The operational advantages of a continuous monitoring system being [44]:

- labor-free measurements;
- accurate data due to real time operational measurements;
- improved service decision due to failure start/progress information;
- unnecessary maintenance reduction due to decisions based on data from continuous measurements;
- prioritization of equipment repair order.

If a full condition monitoring system for switchgear is utilized, such as in [45], it would yield a reduction of 65% in three-year maintenance tasks, increasing the maintenance cycle by 30% and undoubtedly having a positive impact on cost reduction, and ultimately, ensuring a reliable long term switchgear operation. The safety study of ship power failures [46], concluded that in many power failures caused by worn or old components, the root sources were actually deficiencies in the maintenance system. In the case of RMS Neudorf from 2016, the power failure was caused by a breaker malfunction, of which the condition was not monitored regularly or was not noticed during inspection. The process of condition monitoring is the solution to preventing such risks during navigation. In future, the digitalization of switchgear can be utilized in the recent trend of “digital twins” with similar solutions, as described in [47]. Utilization in digital twins is not only limited to switchgear, as the real time operational data can be utilized in the digital twin of a ship; the applications, benefits, and methods of which are revised in [48]. It should be emphasized that the concept of digital switchgear within the digital twin paradigm is of importance in the simulation of electric power systems, since in most studies the complex dynamics of switchgear are often assumed to be irrelevant and circuit breakers are modeled as switches; an example of this can be found in [49]. The implementation of real switchgear operation data will positively impact the overall accuracy of the digital twin, especially in the context of digital twins for ships. Additionally, using effective condition monitoring may reduce maintenance tasks by 65%, saving costs in labor and parts, while also reducing the risk of equipment damage during unnecessary inspections, decreasing failure probability, and optimizing the complete maintenance program [45]. 

### 5.2. Fault Detection

Considering the influence of partial discharges on the development of catastrophic failures, determining the source location (of the defect) is of great importance in maintenance and repair procedures. The implementation of sensors in switchgear automates the detection of faults based on real time operational data obtained through condition monitoring. Confirming that partial discharge has occurred is not enough for failure prevention, unless the location of the discharge is provided in order to determinate the insulation state. One possible solution for defect localization is by utilizing transient earth voltage (TEV) sensors, as in [50], in which the solution is based on the principle that the partial discharge source can be determined by the time difference of arrival method (TDOA) [51]. The TEV method is the optimal solution in switchgear due to its high sensitivity, uninterrupted power supply, strong anti-interference performance, easy installation, and non-instructive method [52]. Some examples of various PD localization methods (including data-driven methods using neural networks) are presented and reviewed in [53,54]. 

### 5.3. Predictive Maintenance

**Figure 5:**
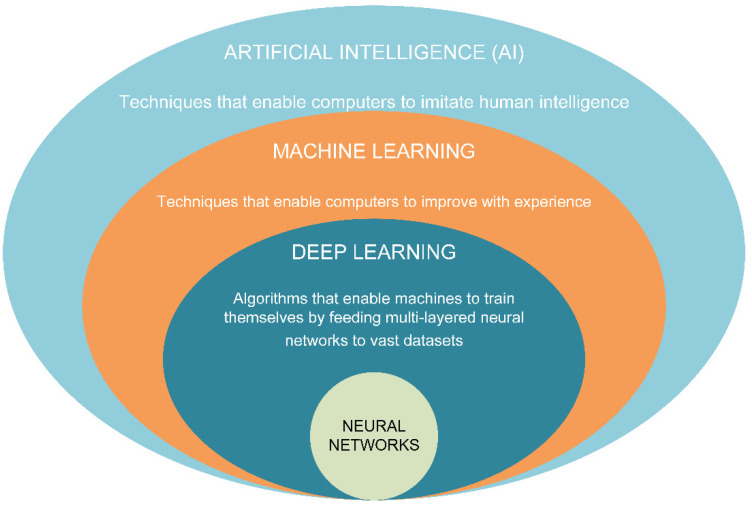
Artificial intelligence divisions illustration.

Considering that predictive maintenance leverages operational data (a collection of various operational parameters over an extended period of time) captured through real time condition monitoring (if implemented) and provides a significant amount of data, the need to automate the recognition, classification, and prediction of potential failures/anomalies is obvious. Until recently, the classification and detection of failures, degradation, or defects have been conducted manually, which had the disadvantage of depending on the expertise of staff. More recent trends use artificial intelligence (AI) algorithms to automate all or part of the data analysis process. In the literature, the terms AI, neural networks, machine learning, and deep learning are often used interchangeably, giving the impression that they are not easily distinguishable. In short, machine learning is a subset of AI, while deep learning is a subset of machine learning, with neural networks being the basis of deep learning, which is distinguished by the number of node layers (also called depth), where a deep learning algorithm must have more than three; the subdivision is shown in Figure 5.

According to [55], the algorithms most commonly used by data scientists in 2021 were linear and logistic regression, followed by decision trees and random forests, while among more complex methods, gradient boosting machines and convolutional neural networks were the most popular approaches. Some examples of the use of AI in switchgear monitoring are presented in Table 1.

There are many more applications of AI, but the common outcome is the detection or prediction of faults or the assessment of the condition of assets, which are then used to make decisions about predictive actions to reduce unwanted downtime in the power grid. In recent years, research has shifted primarily to deep learning (DL) because “traditional” machine learning techniques rely on feature extraction using a predefined set of rules, i.e., rely on the programmer’s accuracy. Deep learning provides a solution to this problem as it can use raw data, and feature selection is integrated within the learning process [67]. A comprehensive review of deep learning in HV applications is given in [68], which also highlights the shortcomings or future needs of deep learning applications. A key challenge is the lack of real-world measurement data, as this is the basis for the development of any AI/ML/DL algorithm. To put it in context: Continuous measurements for temperature monitoring of switchgears are rare or basically non-existent during their entire operation time, and the breaker switching operation is infrequently performed, which makes it extremely difficult to collect data or detect patterns for training algorithms. One solution to the aforementioned problem is the use of a generative adversarial network (GAN), a deep learning algorithm that enables the generation of more data that mimics real data from a limited set of obtained real/experimental data, as in [69]. A full summary of the integration of condition monitoring and machine learning algorithms for predictive maintenance is presented using medium voltage switchgear as an example in [70].

## 6. Discussion

The concept of digital switchgear, as presented in this paper, is an emerging technology, the benefits of which can be seen as a useful addition in monitoring and optimizing power distribution processes, in monitoring the condition of equipment, and as an essential component requiring the implementation of predictive maintenance procedures. Data collection by the various types of implemented sensors will inevitably lead to the establishment of “big data” in distribution networks, which is a compelling step for the full use of various machine/deep learning techniques, as they require large data sets to achieve the desired predictive accuracy. The principles of machine learning/deep learning, or more precisely, their training, are also a major challenge since most techniques are currently trained on data generated either artificially or under laboratory conditions that lack the complex relationships between various parameters in the real world. In the case of [71], the goal was to classify faulty/healthy switchgear using IR images with the additional generation of fake images using generative adversarial networks. Several data generation problems were encountered during the experiments. The experiments revealed that GANs can be used to generate a wide variety of new images, but contribute little to the performance of the algorithm, due to limited variation in the training data. 

Another problem that needs to be emphasized is the impossibility of directly comparing different approaches. There is no question about the accuracy of each study, but it is not necessarily the best standard of comparison, since each method has been validated under different conditions and frameworks. Several studies have compared different approaches using the same data set; they are listed in Table 2.

Following these examples on a global scale, it would be interesting to measure the accuracy of different methods under the same conditions if a well-structured and freely available data set were formed, which would facilitate the tracking of the most recent accuracy achieved and most optimal method. Therefore, future research efforts in both land and marine applications must focus on the creation or acquisition of a freely available database for future analysis to optimize learning algorithms, since fitting each method to a custom database is impractical at this scale. A possible solution to this problem could be to extend or modify the existing COMTRADE file format. COMTRADE is a format for files containing transient waveform and event data (disturbance data that includes voltage, current, power, and frequency) collected by power systems. The format itself is standardized by IEEE C37.111-2013 [72], which is also its main advantage, and consequently, software for file processing has already been developed. Implementation of this format for fault classification and location was achieved in [73], by converting file data into images to train the deep-learning algorithm, and experiments conducted in a laboratory achieved 99.9% accuracy, showing that COMTRADE is a viable solution. Once the challenges are overcome, digital switchgear and predictive maintenance procedures can be combined with the trend of digital twins. Digital twins can help in the design, manufacturing, and testing phases while supporting condition monitoring of switchgear [74]. Furthermore, by creating a digital twin model of a physical device, the database of PD coordinates obtained through simulations can be used to train a localization network, as described in [75], which achieved 90% accuracy and an average localization error of 13.97 cm. If the focus is extended to an entire substation, the digital twin can help with operation, dispatch, management, or a good overall power engineering reference [76]. Altogether, digital switchgear and digital twin technology will certainly have a positive impact on optimizing control processes on ships or onshore, as well as improving overall control strategy and management. As the technology gradually solidifies, standards and procedures (in the maritime domain, the relevant IMO standards) will need to be adapted accordingly to incorporate the technology into established operational procedures and processes.

## 7. Conclusions

This paper describes and analyzes the path of switchgear digitization, divided into past challenges and their solutions, past and current research on their applications, and further divided into condition monitoring, fault detection, and predictive maintenance. The use of machine/deep learning in predictive maintenance is highly appreciated as it enables effective prediction of potential faults by removing the major obstacle of reliance on manual labor for data analysis. The conclusions from this work can be summarized as follows.

1. Since the framework for conducting experimental studies varies, there is an obvious need to “standardize” the procedures, which are also categorized by the type of data used (temperature, partial discharge, voltage/current, etc.). “Standardization” would lead to a universal framework for data collection (creation of a freely accessible database) that could be used to accurately compare the developed algorithms, and adequately monitor improvements in the application of machine/deep learning algorithms in prediction maintenance.
2. The developed dataset could also be used in digital twin simulations of both shore and marine power grids to increase the overall model accuracy, as real data would be implemented, and as the complex dynamics of switchgear are mostly ignored today. Overall, digital twin models offer great optimization opportunities, especially in the maritime domain, which are in line with IMO’s future goals.
3. The development of digital switchgear will lead to an effective predictive maintenance plan that will be continuously optimized with the growing measurement database and machine/deep learning analysis. Repair procedures are optimized, as the source of PD is automatically located, reducing labor and replacement costs. In summary, digital switchgear provides a safer and more cost-effective distribution system compared to its “analog” counterparts. Safety and reliability are enhanced by the active monitoring of equipment to predict failures, and there is a reduction in manpower requirements for data interpretation and equipment monitoring/repair procedures.

As mentioned, the biggest barriers to machine/deep learning adaptation are unbalanced, inconsistent, and limited data sets. The significant lack of data directs research toward generating data that mimics real-world data, but the methods are also inconsistent. Therefore, future work should address the development of a universal dataset, as all studies consequently lack the important step of validation through direct comparison of the proposed methods, which prevents tracking progress and consequently slows it down. The authors future work will include a further investigation of feasible file systems with the aim of solving this issue.

## Figures and Tables

**Table 1:** AI utilization in switchgear monitoring.

Description	Reference
Support Vector Machine (SVM) trained with infrared imagery of substations for equipment monitoring	[56]
Enhancing IR images of rotating machinery and feeding features into SVM and Feed Forward Neural Networks (FFNN) to effectively improve fault diagnosis	[57]
Deep learning-based component recognition in a switchgear overheating fault diagnostic	[58]
PD type identification technology based on deep learning method with a comparison of recognition methods using Convolutional Neural Network (CNN) and Back Propagation Neural Network (BPNN)	[59]
K-Means method to detect partial discharges in equipment	[60,61]
NN and SVM to detect partial discharges in equipment	[62]
NN to detect partial discharge in equipment	[63,64]
Recurrent Neural Network (RNN) to diagnose PD in gas-insulated switchgear	[65]
Deep Convolutional Neural Network (DCNN) to detect PD patterns in gas-insulated switchgear	[66]

**Table 2:** Studies containing direct comparisons.

Reference	Proposed Method	Compared to	Result
[66]	Deep convolutional neural network (DCNN) for PD recognition	Back propagation neural network (BPNN) and support vector machine (SVM)	DCNN has outperformed both BPNN and SVM with 89.7% accuracy
[59]	Residual neural network (ResNet) for PD recognition	BPNN	ResNet outperformed BPNN with 95.83% accuracy (with increased network depth)
[65]	Long short-term memory (LSTM) recurrent neural network (RNN) for PD detection	SVM	LSTM RNN outperformed SVM with 96.74% accuracy

## Data Availability

Not applicable.

## References

[1] World Energy Outlook—Topics. https://www.iea.org/topics/world-energy-outlook.

[2] Bortuzzo V., Bertagna S., Dodero M., Ferrari J., Marinò A., Bucci V. Electrification of Vessels for Garbage Collection and Treatment in Venice Lagoon. Proceedings of the 2021 Sixteenth International Conference on Ecological Vehicles and Renewable Energies (EVER).

[3] Campillo J., Domínguez-Jimenez J.A., Cabrera J. (2019). Sustainable Boat Transportation Throughout Electrification of Propulsion Systems: Challenges and Opportunities. Proceedings of the 2019 2nd Latin American Conference on Intelligent Transportation Systems (ITS LATAM).

[4] 6Anwar S., Zia M.Y.I., Rashid M., de Rubens G.Z., Enevoldsen P. (2020). Towards Ferry Electrification in the Maritime Sector. Energies.

[5] (2020). Fourth Greenhouse Gas Study. https://www.imo.org/en/OurWork/Environment/Pages/Fourth-IMO-Greenhouse-Gas-Study-2020.aspx.

[6] Al-Falahi M., Tarasiuk T., Jayasinghe S., Jin Z., Enshaei H., Guerrero J. (2018). AC Ship Microgrids: Control and Power Management Optimization. Energies.

[7] Chennakeshava R., Niharika Rao R., Karthik M., Mohammed S., Prajath Mahabala R. (2021). A Review on Switchgear Analysis and Common Challenges Observed in Switchgear. IJERT.

[8] Yoon D.-H., Yoon J. (2022). Deep Learning-Based Method for the Robust and Efficient Fault Diagnosis in the Electric Power System. IEEE Access.

[9] Sirojan T., Lu S., Phung B.T., Zhang D., Ambikairajah E. (2022). Sustainable Deep Learning at Grid Edge for Real-Time High Impedance Fault Detection. IEEE Trans. Sustain. Comput..

[10] Gouda O.E., Darwish M.M.F., Mahmoud K., Lehtonen M., Elkhodragy T.M. (2022). Pollution Severity Monitoring of High Voltage Transmission Line Insulators Using Wireless Device Based on Leakage Current Bursts. IEEE Access.

[11] Karandikar H., Neighbours T., Pate R. (2019). The Next Phase in the Evolution of Safety by Design—Digital Switchgear. Proceedings of the 2019 IEEE Petroleum and Chemical Industry Committee Conference (PCIC).

[12] Neighbours T., Moser D. Switchgear Moves into the Digital World. https://library.e.abb.com/public/36631a885cd247b3a968c41504b613fc/Technical%20paper_Digital%20switchgear.pdf.

[13] Deaconu G.G.M., Costinas S., Stoenescu I.B., Opris I. (2021). The Testing of Digital Substation—An Important Issue in Power Engineering Education. Proceedings of the 2021 12th International Symposium on Advanced Topics in Electrical Engineering (ATEE).

[14] Almalaq A., Albadran S., Mohamed M.A. (2022). Deep Machine Learning Model-Based Cyber-Attacks Detection in Smart Power Systems. Mathematics.

[15] Starke A., Nagaraj K., Ruben C., Aljohani N., Zou S., Bretas A., McNair J., Zare A. (2022). Cross-layered Distributed Data-driven Framework for Enhanced Smart Grid Cyber--physical Security. IET Smart Grid.

[16] Elsisi M., Tran M.-Q., Mahmoud K., Mansour D.-E.A., Lehtonen M., Darwish M.M.F. (2021). Towards Secured Online Monitoring for Digitalized GIS Against Cyber-Attacks Based on IoT and Machine Learning. IEEE Access.

[17] Tuballa M.L., Abundo M.L. (2016). A Review of the Development of Smart Grid Technologies. Renew. Sustain. Energy Rev..

[18] Sroubova L., Kropik P., Hamar R., Dolezel I. (2016). Minimization of Force and Thermal Effects in Bus Bars. Proceedings of the 2016 ELEKTRO.

[19] Yusop F.M., Jamil M.K.M., Ishak D., Masri S. (2011). Study on the Electromagnetic Force Affected by Short-Circuit Current in Vertical and Horizontal Arrangement of Busbar System. Proceedings of the International Conference on Electrical, Control and Computer Engineering 2011 (InECCE).

[20] Milovac P., Javora R., Skendzic V. (2017). Sensor Technology in a Medium-Voltage Switchgear for the US Market Applications. CIRED—Open Access Proc. J..

[21] Shepard D.E., Yauch D.W. (2000). An Overview of Rogowski Coil Current Sensing Technology.

[22] Proca V., Paduraru N. (2005). Methods for Non-Conventional Measuring Sensor Integration in the Medium Voltage Electrical Equipment. Proceedings of the 2005 IEEE Russia Power Tech.

[23] Stefanka M., Prokop V., Salge G. (2013). Application of IEC 61850-9-2 in MV Switchgear with Sensors Use. Proceedings of the 22nd International Conference and Exhibition on Electricity Distribution (CIRED 2013).

[24] Elgargouri A., Virrankoski R., Elmusrati M. (2015). IEC 61850 Based Smart Grid Security. Proceedings of the 2015 IEEE International Conference on Industrial Technology (ICIT).

[25] Huang W. (2018). Learn IEC 61850 Configuration in 30 Minutes. Proceedings of the 2018 71st Annual Conference for Protective Relay Engineers (CPRE).

[26] Kumar S., Abu-Siada A., Das N., Islam S. (2021). Toward a Substation Automation System Based on IEC 61850. Electronics.

[27] Al-Tibbi W. (2021). Energy Efficient Software and Hardware Configuration of the Digital Substation in Accordance with IEC 61850. E3S Web Conf..

[28] Subramaniam A., Sahoo A., Manohar S.S., Raman S.J., Panda S.K. (2021). Switchgear Condition Assessment and Lifecycle Management: Standards, Failure Statistics, Condition Assessment, Partial Discharge Analysis, Maintenance Approaches, and Future Trends. IEEE Electr. Insul. Mag..

[29] Slavtchev Y., Mateev V., Tzeneva R. (2019). 3D Coupled Electric-Thermal-Fluid Analysis of Bolted Busbar Connection. Proceedings of the 2019 16th Conference on Electrical Machines, Drives and Power Systems (ELMA).

[30] Park S.W., Cho H. (2014). A Practical Study on Electrical Contact Resistance and Temperature Rise at at the Connections of the Copper Busbars in Switchgears. Proceedings of the 2014 IEEE 60th Holm Conference on Electrical Contacts (Holm).

[31] Budyn M., Karandikar H.M., Urmson M.G. Switchgear Condition Monitoring. 9. CIGRÉ Canada. Proceedings of the Conference on Power Systems.

[32] Woelke B., Monedero M., Jebamony D. Application of Novel Sensor Technology in an Environmental Friendly SF6 Free Medium Voltage Gas Insulated Switchgear Pilot Setup. Proceedings of the VDE High Voltage Technology 2018, ETG-Symposium.

[33] Wildermuth S., Szasz P., Gebhardt J., Kaul H., Koenig K. Infrared Temperature Sensing in Electrical Equipment by Low-Cost IR Cameras. Proceedings of the VDE High Voltage Technology 2018, ETG-Symposium.

[34] Durocher D.B., Loucks D. Infrared Windows Applied in Switchgear Assemblies: Taking Another Look. Proceedings of the 2015 61st IEEE Pulp and Paper Industry Conference (PPIC).

[35] Alam S., Islam T., Mittal U. (2019). A Sensitive Surface Acoustic Wave Sensor for Monitoring Humidity for Substation Application. Proceedings of the 2019 International Conference on Power Electronics, Control and Automation (ICPECA).

[36] Byrne T. Humidity Effects in Substations. Proceedings of the 2014 Petroleum and Chemical Industry Conference Europe.

[37] Meng F., Zhang X., Wu X., Xu B. Experimental Studies on Air Humidity Affecting Partial Discharge in Switchgear. Proceedings of the 2013 Annual Report Conference on Electrical Insulation and Dielectric Phenomena.

[38] Kumpulainen L., Hussain G.A., Lehtonen M., Kay J.A. (2013). Preemptive Arc Fault Detection Techniques in Switchgear and Controlgear. IEEE Trans. Ind. Appl..

[39] Polužanski V., Kovacevic U., Bacanin N., Rashid T.A., Stojanovic S., Nikolic B. (2022). Application of Machine Learning to Express Measurement Uncertainty. Appl. Sci..

[40] Levi D., Gispan L., Giladi N., Fetaya E. (2022). Evaluating and Calibrating Uncertainty Prediction in Regression Tasks. Sensors.

[41] Weigel P., Fischedick M. (2019). Review and Categorization of Digital Applications in the Energy Sector. Appl. Sci..

[42] Esteban A., Zafra A., Ventura S. (2022). Data Mining in Predictive Maintenance Systems: A Taxonomy and Systematic Review. Wiley Interdiscip. Rev. Data Min. Knowl. Discov..

[43] Ayers W., Herinckx T. (2022). Arc Flash in Marine Installations. https://www.ebdg.com/wp-ebdg-content/uploads/2022/02/Arc-Flash-In-Marine-Installations-Technical-Paper-WNA-and-TMH.pdf.

[44] Kane C., Golubev A. Advantages of Continuous Monitoring of Partial Discharges in Rotating Equipment and Switchgear. Proceedings of the Conference Record of the 2003 Annual Pulp and Paper Industry Technical Conference.

[45] Fechet R., Petrariu A.I., Graur A. (2021). Partial Discharge and Internet of Things: A Switchgear Cell Maintenance Application Using Microclimate Sensors. Sensors.

[46] Seatracker (2017). Power Failures on Ships, Safety Study—Safety Investigation Authority.

[47] Helbig D., Singh P., Gomez Hennig E. Transmission Products and Systems for Utilities of the Future—IoT Connected, Digital Twin Based, Intelligent. Proceedings of the 2020 CIGRE Canada Conference.

[48] Assani N., Matić P., Katalinić M. (2022). Ship’s Digital Twin—A Review of Modelling Challenges and Applications. Appl. Sci..

[49] Perabo F., Park D., Zadeh M.K., Smogeli O., Jamt L. (2020). Digital Twin Modelling of Ship Power and Propulsion Systems: Application of the Open Simulation Platform (OSP). Proceedings of the 2020 IEEE 29th International Symposium on Industrial Electronics (ISIE).

[50] Ewaida R.F., Wani N.R., Khan Y., Al-Arainy A.A. (2021). Defect Localization Inside Simulated MV Switchgear Based on Cumulative Energy Curve Using Transient Earth Voltage Sensors. Energies.

[51] Portugues I.E., Moore P.J., Glover I.A., Johnstone C., McKosky R.H., Goff M.B., van der Zel L. (2009). RF-Based Partial Discharge Early Warning System for Air-Insulated Substations. IEEE Trans. Power Deliv..

[52] Yao M. The Application of Temporary Earth Voltage (TEV) Measurement in the Online Monitoring of the Partial Discharge of HV Switch Cabinet. Proceedings of the CICED 2010 Proceedings.

[53] Lu S., Chai H., Sahoo A., Phung B.T. (2020). Condition Monitoring Based on Partial Discharge Diagnostics Using Machine Learning Methods: A Comprehensive State-of-the-Art Review. IEEE Trans. Dielect. Electr. Insul..

[54] Long J., Wang X., Zhou W., Zhang J., Dai D., Zhu G. (2021). A Comprehensive Review of Signal Processing and Machine Learning Technologies for UHF PD Detection and Diagnosis (I): Preprocessing and Localization Approaches. IEEE Access.

[55] (2021). State of Data Science and Machine Learning. https://www.kaggle.com/kaggle-survey-2021.

[56] Rahmani A., Haddadnia J., Seryasat O. (2010). Intelligent Fault Detection of Electrical Equipment in Ground Substations Using Thermo Vision Technique. Proceedings of the 2010 2nd International Conference on Mechanical and Electronics Engineering.

[57] Bai T., Zhang L., Duan L., Wang J. (2016). NSCT-Based Infrared Image Enhancement Method for Rotating Machinery Fault Diagnosis. IEEE Trans. Instrum. Meas..

[58] Zhao K., Li H., Gao S., Li Y., Liu Y., Ma J. (2021). Deep Learning Based Infrared Image Recognize and Internal Overheating Fault Diagnosis of Gas Insulated Switchgear. Proceedings of the 2021 International Conference on Sensing, Measurement & Data Analytics in the era of Artificial Intelligence (ICSMD).

[59] Ding R., Zhao K., Teng Y., Zhuang T., Liu J., Yang J. (2022). Detection and Analysis of GIS Discharge Defects Based on Deep Learninng Method. Proceedings of the 2022 4th Asia Energy and Electrical Engineering Symposium (AEEES).

[60] Peng X., Zhou C., Hepburn D.M., Judd M.D., Siew W.H. (2013). Application of K-Means Method to Pattern Recognition in on-Line Cable Partial Discharge Monitoring. IEEE Trans. Dielect. Electr. Insul..

[61] Lin Y.-H. (2011). Using K-Means Clustering and Parameter Weighting for Partial-Discharge Noise Suppression. IEEE Trans. Power Deliv..

[62] Li L., Tang J., Liu Y. (2015). Partial Discharge Recognition in Gas Insulated Switchgear Based on Multi-Information Fusion. IEEE Trans. Dielect. Electr. Insul..

[63] Si W., Li J., Li D., Yang J., Li Y. (2010). Investigation of a Comprehensive Identification Method Used in Acoustic Detection System for GIS. IEEE Trans. Dielect. Electr. Insul..

[64] Chang C.S., Jin J., Chang C., Hoshino T., Hanai M., Kobayashi N. (2005). Separation of Corona Using Wavelet Packet Transform and Neural Network for Detection of Partial Discharge in Gas-Insulated Substations. IEEE Trans. Power Deliv..

[65] Nguyen M.-T., Nguyen V.-H., Yun S.-J., Kim Y.-H. (2018). Recurrent Neural Network for Partial Discharge Diagnosis in Gas-Insulated Switchgear. Energies.

[66] Song H., Dai J., Sheng G., Jiang X. (2018). GIS Partial Discharge Pattern Recognition via Deep Convolutional Neural Network under Complex Data Source. IEEE Trans. Dielect. Electr. Insul..

[67] Goodfellow I., Bengio Y., Courville A. (2016). Deep Learning.

[68] Mantach S., Lutfi A., Moradi Tavasani H., Ashraf A., El-Hag A., Kordi B. (2022). Deep Learning in High Voltage Engineering: A Literature Review. Energies.

[69] Ardila-Rey J.A., Ortiz J.E., Creixell W., Muhammad-Sukki F., Bani N.A. (2020). Artificial Generation of Partial Discharge Sources Through an Algorithm Based on Deep Convolutional Generative Adversarial Networks. IEEE Access.

[70] Hoffmann M.W., Wildermuth S., Gitzel R., Boyaci A., Gebhardt J., Kaul H., Amihai I., Forg B., Suriyah M., Leibfried T. (2020). Integration of Novel Sensors and Machine Learning for Predictive Maintenance in Medium Voltage Switchgear to Enable the Energy and Mobility Revolutions. Sensors.

[71] Gitzel R., Amihai I., Garcia Perez M.S. (2019). Towards Robust ML-Algorithms for the Condition Monitoring of Switchgear. Proceedings of the 2019 First International Conference on Societal Automation (SA).

[72] IEEE SA—IEEE C37.111-1991. https://standards.ieee.org/ieee/C37.111/2644/.

[73] Hong J., Kim Y.-H., Nhung-Nguyen H., Kwon J., Lee H. (2022). Deep-Learning Based Fault Events Analysis in Power Systems. Energies.

[74] Bhatt A., Karthikeyan V. (2022). Digital Twin Framework and Its Application for Protection Functions Testing of Relays. Proceedings of the 2022 3rd International Conference on Electronics and Sustainable Communication Systems (ICESC).

[75] Miao W., Lingen L., Yong Q., Hui S., Gehao S. (2022). Partial Discharge Inversion Localization Method for GIS Based on Twin Database. Proceedings of the 2022 7th Asia Conference on Power and Electrical Engineering (ACPEE).

[76] He X., Ai Q., Pan B., Tang L., Qiu R. (2022). Spatial-Temporal Data Analysis of Digital Twin. Digit. Twin.

